# Light Pollution Changes the Toxicological Effects of Cadmium on Microbial Community Structure and Function Associated with Leaf Litter Decomposition

**DOI:** 10.3390/ijms21020422

**Published:** 2020-01-09

**Authors:** Zhuangzhuang Liu, Yanna Lv, Rongcai Ding, Xiaxia Chen, Gaozhong Pu

**Affiliations:** 1 School of Pharmacy and Biological Sciences, Weifang Medical University, Weifang 261053, China; huanzjiep52@163.com (Z.L.); dingrc111@163.com (R.D.); 2 Guangxi Key Laboratory of Plant Conservation and Restoration Ecology in Karst Terrain, Guangxi Institute of Botany, Guangxi Zhuang Autonomous Region and Chinese Academy of Sciences, Guilin 541006, China; chenxx7276@163.com

**Keywords:** artificial light at night, litter decomposition, cadmium pollution, extracellular enzyme activities, Illumina Sequencing, microbial biodiversity

## Abstract

Artificial light at night (ALAN/A) can not only alter the behavior and communication of biological organisms, it can also interact with other stressors. Despite its widespread use and the numerous potential ecological effects, little is known about the impact of ALAN on plant litter decomposition under cadmium (Cd) pollution in aquatic ecosystems. In an indoor microcosm experiment, we tested single and combined effects of ALAN and Cd on the activities and community structure of fungi associated with plant litter. The results showed that ALAN and/or Cd can change both water and leaf litter characteristics. ALAN exposure not only altered fungal community structure and their correlations, but also increased the activities of alkaline phosphatase, β-glucosidase, and cellobiohydrolase. The leaf litter decomposition rate was 71% higher in the A-Cd treatment than that in the N-Cd treatment, indicating that the presence of ALAN weakened the negative impact of Cd on leaf litter decomposition. These results suggested that ALAN exposure mitigated the negative effect of Cd on leaf litter decomposition, contributing to the duel effect of ALAN on leaf litter decomposition. Overall, the results expand our understanding of ALAN on the environment and highlight the contribution of ALAN to Cd toxicity in aquatic ecosystems.

## 1. Introduction

Plant litter decomposition plays an important role and provides key ecosystem services through the cycling of organic matter in the freshwater ecosystem governed by microbial decomposers [1]. Among microbial decomposers, fungi have proven to dominate the leaf litter decomposition and are more sensitive to contaminants than bacteria, providing a suitable model to assess contaminant effects on complex ecological systems [2,3].

In freshwater ecosystems, there are more and more emerging pollutants, such as artificial light at night (ALAN, also known as light pollution) because of ongoing anthropogenic activities, especially near the streams there is a high concentration of people [4]. Many studies have indicated that ALAN can not only directly affect a very wide diversity of species including plants, animals, and microbes [4,5,6,7], but also indirectly affect other chemical pollutants by changing their states and associated environmental factors [5,8,9,10]. Interactional effects between natural stressors (temperature, oxygen consumption, and heat) and toxicants (heavy metals and polycyclic aromatic hydrocarbons) have been studied [11,12]. Some recent studies also showed that excessive light (24 h/day) exposure can enhance the inhibitory effect of nanoZnO on explaining light-induced dissolution of nanoZnO [8,9]. Our recent studies have shown that ALAN could not only alleviate the negative effects of silver nanoparticles (AgNP) or Pb on plant litter decomposition in streams but also affect the fungal community composition and function associated with litter decomposition [10,13]. Despite our increasing understanding of the interaction effects of ALAN and chemical pollutants on plant litter decomposition, we still know little about the mechanisms by which microbes adjust their community composition and function in response to ALAN.

Among heavy metals, cadmium (Cd), a non-essential metal element, can cause damage even at very low levels [14]. Currently, Cd pollution is of great concern in both terrestrial and aquatic ecosystems because of human activities. Cadmium, above a certain threshold concentration, is toxic to plants [14,15], animals [16], and microorganisms [17,18,19,20,21]. In freshwater ecosystems, increased cadmium concentrations can inhibit fungal reproduction and diversity [19] and closely related to water chemistry [20]. Simultaneously, Cd stress can cause some cadmium-resistant species to help maintain ecosystem processes [21]. Several studies have demonstrated that Cd alone or combined with phenanthrene or high temperature can inhibit the growth, reproduction, and negatively affect the diversity and activity of fungi depressing plant-litter decomposition in streams [12,19,20,21,22,23]. Although concern about the potential ecological impacts of Cd and/or some natural stressors has long been known, the combined effects of Cd and/or ALAN on leaf litter decomposition and associated fungal communities remain difficult to predict despite their important role in freshwater ecosystems.

The present study aimed to investigate the interactive effects of ALAN and/or Cd on leaf litter decomposition in freshwater ecosystems. We hypothesized that fungal community structure and enzyme activities are modulated by ALAN and/or Cd. We expected that the changes caused by fungal structure and metabolic activities will affect the subsequent litter decomposition. To test this hypothesis, *Pterocarya stenoptera* C. DC. leaf litter decomposition rates and associated fungal communities, microbial biomass, and metabolic activities were measured under Cd stress in two different light conditions in an indoor microcosm experiment.

## 2. Results

### 2.1. Stream Water Chemistry

The physical and chemical characteristics of the stream water used in the microcosms were significantly different among treatments at the end of the experiment (Table 1). Compared with the control (N treatment), the ALAN treatment resulted in significant increases in pH value, dissolved oxygen andNH_4_^+^ concentration (Table 1). Especially ALAN and/or Cd exposure always resulted in a significant decrease in total suspended solids compared with the control (N treatment). Furthermore, the presence of ALAN also decreased the turbidity and total suspended solids, but increased the conductivity of the stream water.

### 2.2. Fungal Communities

**Figure 1:**
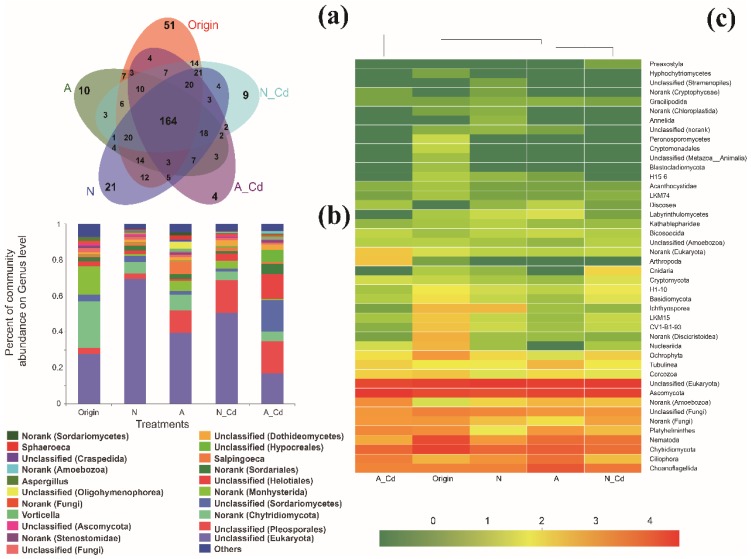
Fungal taxonomic composition by operational taxonomic units (OTUs) (**a**) and genus (**b**) levels and the most dominant 30 genera heat map diagram (**c**) among different treatments. N, natural light simulation group without cadmium; A/ALAN, simulation group without cadmium; N-Cd, natural light simulation group with cadmium; N-Cd, ALAN simulation group with cadmium.

**Figure 2:**
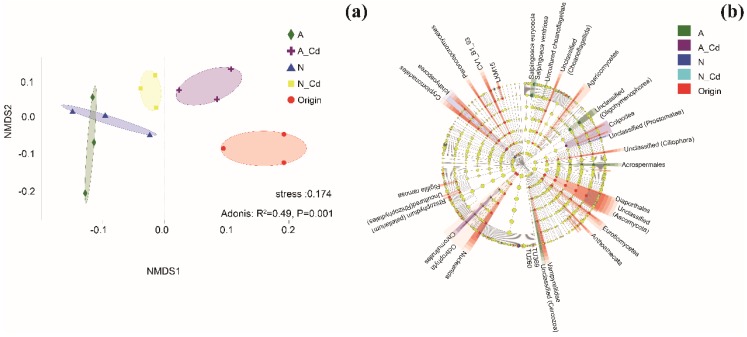
Multidimensional scaling (NMDS) ordination (**a**) and linear discriminant analysis effect size (LEfSe) cladogram (**b**) of fungal communities among the different treatments and origin. Treatment abbreviations are defined in Figure 1.

Sequence composition analyses showed that there were significant differences in the number of operational taxonomic units (OTUs) and the Simpson index between the control and the other treatments (Table A1). Compared with the control, the ALAN and/or Cd exposure always decreased the unique fungal OTUs (Figure 1a) and altered the abundance of the six most abundant genera, but the combination of Cd and ALAN responded much more to this abundance change than its individual effect (Figure 1b). For example, the relative abundance of an unclassified genus of Eukaryota was three-fold higher in the N-Cd treatment than in the A-Cd treatment, while the relative abundance of an unclassified genus of Sordariomycetes was 11-fold higher in the A-Cd treatment than in the N-Cd treatment. In addition, the relative abundance of an unclassified genus of Pleosporales was 4.6-, 6.8-, and 6.7-fold higher in the ALAN treatment, the N-Cd treatment, and the A-Cd treatment than in the control. The heat map analysis shows that the compositions of the top 30 dominant fungal phyla in the A-Cd treatment were different from the other treatments and the origin (Figure 1c). Multidimensional scaling (NMDS) analysis revealed that the fungal communities from the A-Cd treatment, the N-Cd treatment, and the origin were clearly separated from other treatments (Figure 2a).

To better visualize and explore the data, linear discriminant analysis effect size (LEfSe) analysis was carried out. The results revealed that each treatment (except the N-Cd treatment) had its own fungal indicator taxa, from class to genus level (Figure 2b). For example, Nucleariida, Vampyrellidae, Eurotiomycetes, Ascomycota, Agaricomycetes, Peronosporomycetes, and Cryptomonadales were enriched in the original samples; Ichthyosporea and an unclassified genus (Prostomatea) were enriched in the N treatment; Acrospermales, *Salpingoecaeuryoecia*, an unclassified class (Cercozoa) and species (Oligohymenophorea), and uncultured choanoflagellate were enriched in the ALAN treatment; *Salpingoecaventriosa* was enriched in the N-Cd treatment; Colpodea and Chromulinales were enriched in the A-Cd treatment.

**Figure 3:**
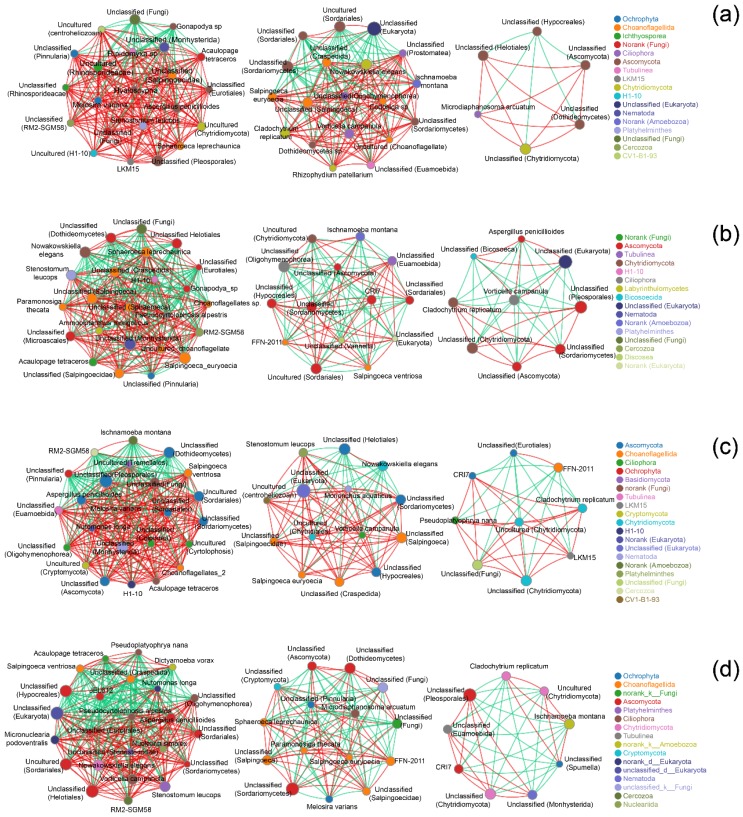
Co-occurring fungi at the species level in the control (N treatment) (**a**), artificial light at night (ALAN) treatment (**b**), N-Cd treatment (**c**), and A-Cd exposure (**d**). Sizes and colors of the nodes represent the relative abundance of the fungi. Solid lines in red and green denote positive and negative correlations, respectively. The width reflects the strength of the correlation.

Correlation network analysis indicated that although the fungi associated with litter decomposition in different treatments resulted in analogous patterns of fungal clustering, the species and the strengths of the correlations differed (Figure 3). In an analysis of 50 fungi at the species level, there were 332 positive and 96 negative correlations in the control (Figure 3a), 279 positive and 124 negative correlations in the N-Cd treatment (Figure 3b), 235 positive and 169 negative correlations in the ALAN treatment (Figure 3c), and 225 positive and 187 negative correlations in the A-Cd treatment (Figure 3d). For example, the most abundant species (an unclassified member of Eukaryota) is negatively correlated with 15 species and positively correlated with three unclassified members of Ascomycota which clustered within the control (Figure 3a), but negatively correlated with 5, 2, and 5 species and positively correlated with 3, 11, and 16 different species in the ALAN treatment, the N-Cd treatment, and A-Cd treatment, respectively (Figure 3b–d).

### 2.3. Changes in Extracellular Enzyme Activities

**Figure 4:**
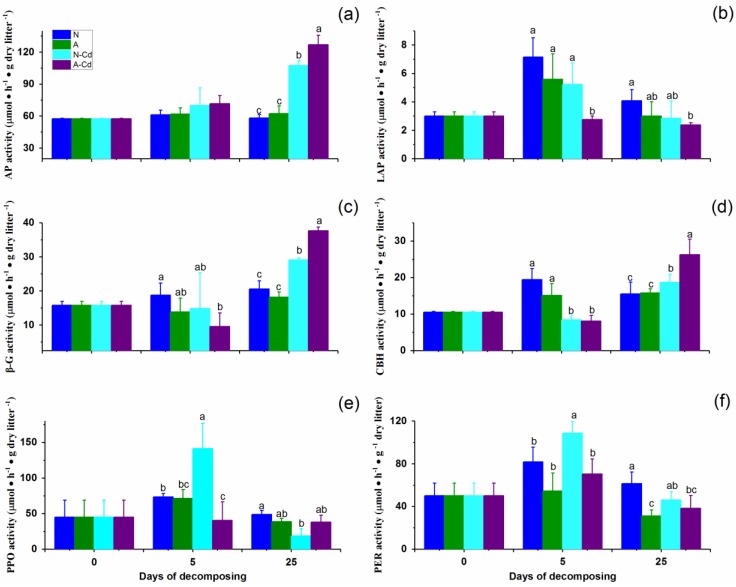
Changes in the enzymatic activities of leaf litter during the decomposition process. Legends: (**a**) acid phosphatase, AP; (**b**) leucine-aminopeptidase, LAP; (**c**) β-glucosidase, β-G; (**d**) cellobiohydrolase, CBH; (**e**) polyphenol oxidase, PPO; (**f**) phenol oxidase, PER; N: Natural lighting simulation group; A: artificial light at night simulation group. Different letters denote a significant difference between treatments at the same sampling time (*p* < 0.05; Tukey′s test).

**Figure A1:**
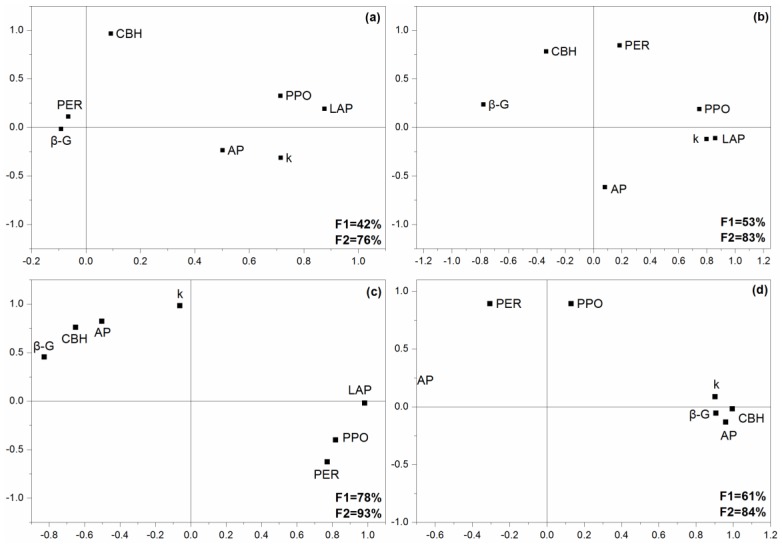
Principal component analysis of the correlation patterns of enzymatic activities and litter decomposition rate (k) in the natural light simulation group without cadmium (N) (**a**), ALAN simulation group without cadmium (A) (**b**), natural light simulation group with cadmium (N-Cd) (**c**), and ALAN simulation group with cadmium (A-Cd) (**d**). AP, acid phosphatase; β-G, β-Glucosidase; CBH, cellobiohydrolase; LAP, leucine-aminopeptidase; PPO, polyphenol oxidase; PER, peroxidase.

Extracellular enzyme activity significantly varied with exposure time, the treatments, and the interaction between the two factors (two-way ANOVA, *p* < 0.05). After 25 days, Cd exposure significantly increased the activities of acid phosphatase (AP), β-glucosidase (β-G), and cellobiohydrolase (CBH), while compared with the N-Cd treatment, A-Cd treatment significantly increased the activities of AP, β-G, and CBH (Figure 4). Principle component analysis (PCA) showed that the A-Cd treatment enhanced the correlations between the litter decomposition rates and the activities of AP, β-G, and CBH compared with the N-Cd treatment. Exposure of only ALAN enhanced the correlations between the litter decomposition rates and the activities of leucine aminopeptidase (LAP), polyphenol oxidase (PPO), and phenol oxidase (PER) compared with the control (Figure A1).

### 2.4. Microbial Biomass

**Figure 5:**
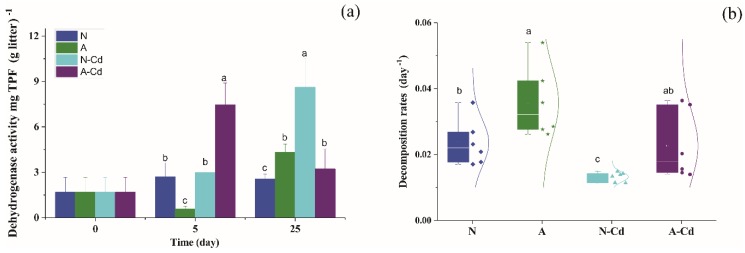
Decomposition rate of *Pterocarya stenoptera* leaf litter (**a**) and associated dehydrogenase activity (**b**) after 25 days of incubation in the microcosms. Legend: N, natural light simulation group without Cd; A, artificial light at night (ALAN) simulation group without Cd; N-Cd, natural light simulation group with Cd; N-Cd, ALAN simulation group with Cd. Different lowercase letters on top of the bars denote significant differences (*p* < 0.05) among the treatments.

The microbial biomass, determined by dehydrogenase activity (DHA), was affected by both Cd concentration and exposure time (two-way ANOVA, *p* < 0.05). The ALAN alone exposure significantly decreased the DHA but the A-Cd treatment significantly increased DHA (Figure 5a) after five days of incubation. However, the N-Cd treatment significantly increased DHA after 25 days of incubation.

### 2.5. Leaf Chemical Characteristics and Mass Loss

Compared with the control (the N treatment), the ALAN treatment significantly increased the decomposition of *P. stenoptera* leaf litter (Figure 5b). However, compared with the other treatments, the N-Cd treatment significantly decreased litter decomposition rates (*p* < 0.05). The leaf litter decomposition rate was 71% higher in the A-Cd treatment than in the N-Cd treatment. Compared with the control, the A-Cd treatment increased the carbon and phosphorus contents, and the N-Cd treatment increased the nitrogen content (Table 2). The ALAN treatment significantly decreased the lignin content of leaf litter (Table 2).

## 3. Discussion

This study evaluated the combined effects of an emerging pollutant (ALAN) and heavy metal (Cd) pollutant on the community structure and metabolic activities of fungi associated with plant litter decomposition in a stream ecosystem. An important result shown by this research is that ALAN exposure mitigates the negative effect of Cd on leaf litter decomposition. Our results also provide strong evidence for the importance of considering emerging environmental parameters when assessing the potential risks of heavy metals to freshwater biota and ecosystem processes.

Fungi, associated with litter decomposition, play key roles in the carbon and nutrient dynamics of stream ecosystems and are more sensitive to pollutants than bacteria [3]. Hence, the fungal community can be used as an indicator to evaluate the response to environmental stress [24]. Previous studies showed that light (UV radiation or ALAN) cannot only increase the degradation of complex aromatic compounds [25], but also alter microbial communities [5,8,9,10]. Our results showed that ALAN exposure always decreased unique fungal OTUs and altered the abundance of the six most abundant genera (Figure 1a,b). Furthermore, NMDS analyses showed that A-Cd exposure clearly separated from the other treatments, and the fungal community composition at the OTU level was significantly different (ANOSIM: R = 0.49, *p* = 0.001). These results indicated that ALAN exposure can alter the fungal community structure and composition. This is consistent with the results of recent studies which showed that light radiation not only can alter the fungal community, but also inhibit litter decomposition associated with fungi [8,9,10]. However, our results differ from these studies [8,9] in that effects of different light conditions on the structure and diversity of fungal communities are significantly different. In Du’s studies [8,9], the leaves were incubated under visible light (24 h/day) which would almost completely change the living environment of the fungi and the samples were not replicated when measuring fungal diversity. However, in the present study, the leaves were incubated under a mixture of artificial light (night, 12 h) and natural light (day, 12 h) which may be more similar to a natural environment under ALAN stress and three replicated samples were collected in each treatment. For example, in our study, ALAN exposure always increased the abundance of Ascomycota, Chytridiomycota, and Tubulinea, but decreased the abundance of Ochrophyta, h1-10, and LKM15, regardless of Cd presence (Figure 1c). This indicated that ALAN may be the main controlling factor leading to the change in fungal community. In addition, our results also showed that although the fungi associated with litter decomposition in different treatments resulted in analogous patterns of fungal clustering, the species and the strengths of the correlations differed (Figure 3), indicating that ALAN can also change their correlations and functions. In summary, we can conclude that ALAN exposure not only altered the fungal community structure and composition, but also changed their correlations and functions.

Previous studies have shown that a shift in community composition due to the replacement of sensitive species by tolerant ones is a common response to contaminated ecosystems [3,26,27]. During leaf litter decomposition, some fungi (aquatic hyphomycetes) are very efficient at producing metal-binding proteins, which allow them to tolerate some degree of metal contamination [27]. Our LEfSe analysis showed that Acrospermales and *Salpingoecaeuryoecia* spec. nov. were enriched in the ALAN treatment, *Salpingoecaventriosa* spec. nov. was enriched in the N-Cd treatment, and Colpodea and Chromulinales were enriched in the A-Cd treatment, suggesting that these fungi may be ALAN and/or Cd resistant fungi that are involved in adaptive mechanisms toward tolerances against ALAN and Cd stress. This finding supported the hypothesis that the biological characteristics of the altered microbial community facilitated the ability of the community to cope with Cd toxicity [3]. Interestingly, this is consistent with previous findings that leaf litter decomposition related fungi could form adaptive mechanisms for tolerance to NPs and/or metal ions (e.g., ZnONP, AgNP, and Cu^2+^) by accumulating dominant species [3,10,28,29].

An important observation from our results is that ALAN exposure mitigates the negative effect of Cd on leaf litter decomposition. Our results showed that the leaf litter decomposition rate was 71% higher in the A-Cd treatment than in the N-Cd treatment, indicating that the presence of ALAN weakened the negative impact of Cd on leaf litter decomposition rates by affecting microorganisms associated with litter decomposition. One possible reason may be that accelerated degradation of leaf lignin under ALAN exposure since photo degradation can release carbon from lignin and increase the accessibility of many other compounds locked in lignin linkages within the cell walls to biotic degradation [30,31,32]. Our results showed that the ALAN treatment significantly decreased the lignin content of leaf litter, suggesting ALAN exposure can increase litter decomposition by increasing the microbial accessibility to lignin [33]. Another reason may be that ALAN exposure can decrease the toxicity of Cd on leaf litter decomposition by altering the microbial composition and activities associated with litter decomposition. Our results showed that ALAN exposure altered the fungal community structure and their correlations and functions, and developed some ALAN and/or Cd stress resistant fungi. This suggests that litter-related fungi may affect the degradation process of leaf litter by altering the structure, composition, and correlation of fungal communities. Previous studies showed that changes in fungal community is one of the main factors affecting the change in litter decomposition [27,34,35,36]. Our results also showed that compared with the N-Cd treatment, the A-Cd treatment significantly increased the activities of AP, β-G, and CBH and enhanced the correlations between the litter decomposition rates and these enzymatic activities. This indicates that ALAN exposure alleviates the inhibition effect of Cd on the plant litter decomposing process by accelerating the conversion of carbon and phosphorus since β-G and CBH play significant roles in carbon cycling, and AP in phosphorus cycling [37].

## 4. Materials and Methods 

### 4.1. Leaf Conditioning

The study was conducted in a first-order hard-water stream of Lijiang River in the southeastern lowlands of China (25°51′13.5″N, 110°24′59.1″E, and an altitude of 433 m). The dominant species near the stream was *P. stenoptera*. *P. stenoptera* leaves were collected in September 2017 by binding nylon nets on the trees along the Lijiang River and air dried at room temperature. Leaves were cut into disks (14-mm diameter), enclosed in fine mesh bags (18 × 25 cm, 0.5 mm mesh size) that were immersed in the Lijiang River from 25 October to 5 November for microbial colonization. During leaf immersion, physical and chemical characteristics of the stream water were measured in situ by a SEBA MPS-Checker (SEBA Hydrometrie, Kaufbeuren, Germany) and results are shown in Table A2. Additionally, 40 L of stream water was collected from the colonized stream and stored in a refrigerator (4 °C) for water renewal throughout the whole experiment.

### 4.2. Microcosm Experiment

After soaking, the microbially colonized leaf discs were placed into sterile Erlenmeyer flasks (150 mL) with 80 mL of the filtered (Whatman^®^, 1.2 mm pore size) and sterilized (121 °C, 30 min) stream water. Four treatments were prepared for microcosms of litter decomposition: (1) N, litter was incubated in a natural light simulation group (natural light: dark/12 h: 12 h) without Cd addition, (2) N-Cd, leaves were incubated in a natural light simulation group with Cd (CdCl_2_·2.5H_2_O, 1000 μg·L^-1^), (3) A/ALAN, leaves were incubated in an ALAN simulation group (natural light: artificial light /12 h: 12 h) without Cd, and (4) N-Cd, leaves were incubated in an ALAN simulation group with Cd (CdCl_2_·2.5H_2_O, 1000 μg·L^-1^). To simulate ALAN, two small LED lights (8 W, NVC 6500 K, Zhejiang, China) with a mean illumination of 180 ± 13 lux at night were prepared. In the natural light simulation group, the mean illumination was 535 ± 48 during the day and 0.11 ± 0.03 lux at night. All microcosms were shaken at 150 rpm under 18 °C for 25 days, and water was renewed every 5 days (including CdCl_2_·2.5H_2_O). After 5 and 25 days of incubation, sets of 12 microcosms (three replicates for each treatment per time) were immediately sampled to determine pH value, remaining leaf mass, lignin, carbon and nitrogen content, fungal diversity, microbial biomass, as well as extracellular enzyme activity.

### 4.3. Leaf Mass Remaining, Lignin, Carbon, and Nitrogen Content

Remaining leaf mass was assessed as described by Pu et al. [30] and leaf decomposition rates (k) were calculated according to Olson [38]. The lignin content of leaf litter was determined using a gravimeter via hot sulfuric acid digestion following the method of Gessner [39]. The content of leaf litter nitrogen and carbon were determined using an elemental analyzer (Elementar Analysensysteme, Langenselbold, Germany).

### 4.4. Fungal Diversity

Illumina MiSeq sequencing technology was adopted to evaluate changes in the fungal diversity and community structure associated with litter decomposition. The DNA of each litter sample was extracted and purified using an E.Z.N.A. Plant DNA Kit (OMEGA Bio-Tek, Norcross, GA, USA) and 1% agarose gel electrophoresis, respectively. The ITS regions of the fungal18S rDNA genes were amplified using the universal primers SSU0817F (5ʹ-TTAGCATGGAATAATRRAATAGGA-3ʹ) and 1196R (5ʹ-TCTGGACCTGGTGAGTTTCC-3ʹ). The PCR analysis was performed in the following sequence: 94 °C, 3 min followed by 5× (94 °C, 30 s; 45 °C, 20 s; 65 °C, 30 s) and 25× (94 °C, 20 s; 55 °C, 20 s; 72 °C, 30 s), and a final extension at 72 °C for 5 min. Illumina MiSeq sequencing was performed using an Illumina MiSeq platform (Illumina, San Diego, CA, USA) according to the standard protocols by Majorbio Bio-Pharm Technology Co., Ltd. (Shanghai, China). The sequences were clustered into operational taxonomic units (OTUs) at 97% similarity threshold using UPARSE (version 7.1 http://drive5.com/uparse/). Then the Uchime algorithm was used to detect chimeric sequences [40]. The Ribo-somal Database Project pipeline (RDP) (http://pyro.cme.msu.edu/) was used to classify and analyze each 18S rRNA gene sequence. The sequence reads with an average quality score of <25 and with a read length of <200 bp were removed after trimming off the last 30 bps, and ambiguous bases and homo-polymers of <8 bases were also removed from the dataset. Finally, the complete dataset was sent to the Sequence Read Archive (SRA) database of the National Center for Biotechnology Information (NCBI) under the accession numbers of SRP238830 for fungi.

### 4.5. Microbial Biomass

Microbial biomass was evaluated by dehydrogenase activity (DHA) since it is directly involved in the respiratory chain [22]. To compare the changes in microbial biomass, three disks with 0.4 mL of triphenyltetrazolium chloride (TTC) solution (0.1 g of TTC per 100 mL of 100 mM Tris buffer, pH of 7.6) were incubated in the dark at 25 °C for 24 h. Acetone (4 mL) was then added to each sample, followed by a 2 h incubation period (25 °C). The absorbance of the solutions was measured at 485 nm by using a spectrophotometer (PerkinElmer AA 600, Waltham, MA, USA). Three autoclaved disks of each sample were analyzed as the negative controls.

### 4.6. Extracellular Enzyme Activities

The enzymatic activities associated with leaf litter decomposition involved in nitrogen (leucine-aminopeptidase, LAP), phosphorus (alkaline phosphatase, AP), and carbon (β-glucosidase, β-G; cellobiohydrolase, CBH) cycling and polyphenol metabolism (peroxidase, PER;polyphenol oxidase, PPO) were assessed by colorimetric assays according to the methods described in the Allison Lab Protocol [41].

### 4.7. Data Analysis

Statistically significant differences among various treatments were analyzed using SPSS (version 18.0). Alpha and beta diversity metrics were computed within QIIME (weighted Uni-Frac distance, http://qiime.org/index.html) [42]. The fungal community composition over all samples was analyzed using nonmetric multidimensional scaling (NMDS). Significant taxonomic differences between the five different treatments were performed using the linear discriminant analysis effect size (LEfSe) method (http://huttenhower.sph.harvard.edu/galaxy/root). To better understand the fungal interactions within a community and their responses to different treatments, a co-occurrence network analysis was carried out using the free online platform of Majorbio I-Sanger Cloud Platform (www.i-sanger.com).

## 5. Conclusions

In conclusion, one important ecological effect of our results is that ALAN exposure mitigates the negative effect of Cd on leaf litter decomposition, this can be explained by the decreased lignin content of leaf litter, altered fungal community structure and the correlation between fungal species, and the changes in enzyme activities. This finding underpins the importance of considering environmental parameters and provides new horizons for ecological safety assessment of heavy metal pollutants in freshwater ecosystems.

## Figures and Tables

**Table 1:** Hydrographical and chemical characteristics of stream waters during leaf immersion and decomposition in microcosms.

Treat.	pH	DO (mg L^–1^)	NH_4_^+^ (ppm)	NTU	TDS (mg L^–1^)	TSS (mg L^–1^)	Cond. (μs cm^–1^)
Origin	6.80 ^c^	5.35 ^a^	42 ^c^	421 ^b^	24.333 ^a^	2.000 ^b^	0.036 ^c^
N	7.38 ^b^	4.61 ^d^	56 ^b^	595 ^a^	0.011 ^b^	2.389 ^a^	0.017 ^d^
A	7.53 ^a^	4.75 ^d^^b^	77 ^a^	671 ^a^	0.011 ^b^	0.003 ^e^	0.016 ^d^
N_Cd	7.39 ^b^	4.70 ^bc^	60 ^b^	136 ^d^	0.082 ^b^	0.542 ^d^	0.122 ^b^
A_Cd	7.33 ^b^	4.65 ^cd^	62 ^b^	290 ^c^	0.083 ^b^	1.171 ^c^	0.125 ^a^

Note: Treat. = treatments; DO = dissolved oxygen; NTU = turbidity; TDS = total dissolved solids oxygen; TSS = total suspended solids; Cond. = conductivity; N, natural lighting simulation group; A, artificial light at night simulation group. Different lower letters denote significant differences between treatments (*p* < 0.05).

**Table 2:** Carbon, nitrogen, phosphorus, and lignin contents presented as milligram gram of dry litter mass.

Treatment	Carbon mg g^–1^	Nitrogen mg g^–1^	Phosphorus (mg g^–1^)	Lignin (%)
N	286.73 ^b^	25.29 ^b^	1.32b ^c^	5.10 ^a^
A/ALAN	348.04 ^b^	26.48 ^ab^	1.28 ^c^	2.21 ^b^
N_Cd	327.29 ^b^	30.81 ^a^	1.48 ^ab^	6.45 ^a^
A_Cd	455.59 ^a^	26.71 ^ab^	1.60 ^a^	2.82 ^b^

Note: N, natural lighting simulation group; A/ALAN, artificial light at night simulation group. Different lower letters denote significant differences between treatments (*p* < 0.05).

## Appendix A

**Table A1:** Abundance and diversity of fungal and bacterial communities associated with the litter decomposition.

Samples	Sequence	OTUs	Sobs	Shannon	Simpson	ACE	Chao1
Origin	46,086 ^a^	365 ^a^	284 ^a^	3.10 ^a^	0.15b ^c^	314 ^a^	314 ^a^
N	41,864 ^b^	321 ^b^	215 ^ab^	2.04 ^b^	0.37 ^a^	243 ^ab^	249 ^ab^
A	51,507 ^b^	285 ^c^	198 ^b^	2.62 ^ab^	0.21 ^bc^	222 ^b^	220 ^b^
N_Cd	41,698 ^ab^	307 ^c^	218 ^b^	2.30 ^b^	0.28 ^b^	248 ^b^	242 ^b^
A_Cd	40,860 ^b^	274 ^c^	171 ^b^	2.49 ^ab^	0.18 ^d^	194 ^b^	195 ^b^

Note: N, natural lighting simulation group; A, artificial light at night simulation group. Different lower letters denote significant differences between treatments (*p* < 0.05).

**Table A2:** Hydrographical and chemical characteristics of stream waters during leaf immersion.

Par.	PH	DO (mg L^–1^)	Cond. (μs cm^–1^)	Sal	NTU	OPR (mv)
water	6.78	4.81	0.036	0.017	0.44	160
Par.	TSS (mg·L^–1^)	DOC (mg·L^–1^)	TP	NH_4_^+^ (ppm)	T (°C)	TN
water	3.01	1.08	0.042	0.79	20.41	1.73
Par.	Chla (μg·L^–1^)	TDS (mg·L^–1^)	Ca^2+^ (mg·L^–1^)	Mg^2+^ (mg·L^–1^)	Cd^2+^ (μg·L^–1^)	
water	0.77	24	68	4.56	<0.001	

Par. = Parameters; DO = dissolved oxygen; Cond = conductivity; Sal = salinity; TSS = total suspended solids; DOC = dissolved organic carbon; TN = total nitrogen; TP = total phosphorus; Chl-a = chlorophyll-a; NTU = turbidity; OPR = oxidation reduction potential; TDS = total dissolved solids oxygen; T = temperature.
